# De novo genome assembly and analysis unveil biosynthetic and metabolic potentials of *Pseudomonas fragi* A13BB

**DOI:** 10.1186/s12863-021-00969-0

**Published:** 2021-05-18

**Authors:** Opeyemi K. Awolope, Noelle H. O’Driscoll, Alberto Di Salvo, Andrew J. Lamb

**Affiliations:** 1 School of Pharmacy and Life Sciences, Robert Gordon University, Sir Ian Wood Building, Garthdee Road, Aberdeen, AB10 7GJ Scotland; 2 Graduate School, Robert Gordon University, The Ishbel Gordon Building, Garthdee Road, Aberdeen, AB10 7QE Scotland

**Keywords:** *Pseudomonas fragi*, β-Lactone antibiotics, Plant growth-promoting rhizobacteria, Rhizosphere microbiome

## Abstract

**Objectives:**

The role of rhizosphere microbiome in supporting plant growth under biotic stress is well documented. Rhizobacteria ward off phytopathogens through various mechanisms including antibiosis. We sought to recover novel antibiotic-producing bacterial strains from soil samples collected from the rhizosphere. *Pseudomonas fragi* A13BB was recovered as part of this effort, and the whole genome was sequenced to facilitate mining for potential antibiotic-encoding biosynthetic gene clusters.

**Data description:**

Here, we report the complete genome sequence of *P. fragi* A13BB obtained from de novo assembly of Illumina MiSeq and GridION reads. The 4.94 Mb genome consists of a single chromosome with a GC content of 59.40%. Genomic features include 4410 CDSs, 102 RNAs, 3 CRISPR arrays, 3 prophage regions, and 37 predicted genomic islands. Two β-lactone biosynthetic gene clusters were identified; besides, metabolic products of these are known to show antibiotic and/or anticancer properties. A siderophore biosynthetic gene cluster was also identified even though *P. fragi* is considered a non-siderophore producing pseudomonad. Other gene clusters of broad interest identified include those associated with bioremediation, biocontrol, plant growth promotion, or environmental adaptation. This dataset unveils various un−/underexplored metabolic or biosynthetic potential of *P. fragi* and provides insight into molecular mechanisms underpinning these attributes.

## Objective

The rhizosphere has been described as one of the most complex ecosystems on Earth, harboring abundant dynamic plant-microbe and microbe-microbe interactions. Plant growth-promoting rhizobacteria (PGPR) are one of the components of this ecosystem where they promote plant growth by enhancing uptake of nutrients and inorganic elements, or by increasing resistance to various environmental stresses including heavy metals, high salt concentrations and phytopathogens [1, 2]. PGPR protect against phytopathogens through a variety of mechanisms, including the ability to gain competitive advantage for nutrients and trace elements and/or produce one or more antibiotics effective against such pathogens [1, 2]. Whilst the latter characteristic (which is common to many soil dwelling bacteria) has been exploited to develop many clinically useful antibiotics, it remains the case that less than 1% of all known bacterial species have had their metabolic capabilities exploited in this way [3]. We therefore sought to recover potential novel antibiotic-producing bacterial strains from soil samples collected from the rhizosphere of various plants. *Pseudomonas fragi* strain A13BB was isolated as part of this effort.

*P. fragi* is a Gram-negative, rod-shaped, aerobic psychrophile. It is widely distributed in nature and commonly associated with meat and dairy spoilage [4, 5]. It is rarely reported as a PGPR except by Selvakumar et al [5] and Fahr et al [6] who reported its phosphate solubilisation activity and its ability to improve tolerance against aluminium stress in acidic soils, respectively. However, to the best of our knowledge, it has not been previously reported as an antibiotic producer. Therefore, being a species not readily associated with antibiotic production, the genome of *P. fragi* A13BB was sequenced to facilitate mining for potential antibiotic-encoding secondary metabolite biosynthetic gene clusters (smBGCs) and other gene clusters that may be responsible for its environmental adaptation and plant growth promotion.

## Data description

*P. fragi* A13BB was isolated from the rhizosphere of a plant in Aberdeen, Scotland (57.101 N 2.078 W) using an ultra-minimal substrate medium (data file 1) [7]. Purified strain was cultivated in nutrient broth (Oxoid, UK) at 28 °C for 24 h before gDNA was extracted from pellets with the DNeasy® Ultraclean® Microbial Kit for DNA Isolation (Qiagen, UK). The extract was used as template to amplify the 16S rRNA gene in PCR reactions using 27F and U1510R universal primers, with thermocycler parameters set as follows: Initial denaturation at 95 °C for 2 min followed by 30 cycles of further denaturation at 95 °C for 30 s, primer annealing at 45 °C for 30 s and elongation at 72 °C for 105 s. A final elongation was carried out at 70 °C for 5 min. Amplified DNA fragment was sequenced using the 27F primer. Isolate was subsequently identified by 16S rRNA gene comparison as *P. fragi* with 99% identity score.

Libraries were prepared for Illumina sequencing by Glasgow Polyomics (Glasgow, UK) using the Nextera XT DNA Library Preparation Kit (Illumina, USA) following manufacturer’s protocol, and sequenced with the Illumina MiSeq using a 300 bp paired end protocol. Libraries were prepared for GridION sequencing by MicrobesNG (Birmingham, UK) using the Oxford nanopore SQK-RBK004 kit and/or SQK-LSK109 kit with Native Barcoding EXP-NBD104/114 (ONT, UK), and sequenced on a FLO-MIN106 (R.9.4 or R.9.4.1) flow cell in a GridION (ONT, UK).

Illumina reads were trimmed with Trimmomatic [8] v0.36 operated in the sliding window mode with Q25 quality cut-off and minimum read length of 100. The quality of trimmed reads was assessed with FastQC [9] v0.11.8 and results were aggregated with MultiQC [10] v1.8 (data file 2) [11]. Mean quality score across each base position was ≥31. Quality assessment of GridION reads was performed with NanoPlot [12] v1.28.2. Quality statistics are summarised in data file 3 [13], while average read quality plot is displayed in data file 4 [14].

Paired short reads and long reads were assembled de novo with Unicycler [15] v0.4.8. Assembly quality was assessed with Quast [16] v5.0.2. Two contigs were identified (data file 5) [17], the smaller contig (5386 bp) representing the complete genome of bacteriophage φX174 (control spike in Illumina sequencing) was subsequently extracted from the data. The larger contig (4,940,458 bp) represents the complete genome of *P. fragi* A13BB with sequencing depths of 226x and 32x for Illumina and GridION sequencing, respectively. Assembly completeness was 99.2% as assessed with BUSCO [18] v4.1.2 using the pseudomanadales_odb10 lineage dataset (data file 6) [19]. Assembly graph was visualised with Bandage [20] and displayed in data file 7 [21]. ANI analysis with the FastANI tool [22] v1.3 confirmed identity as *P. fragi* with the ANI value of 98.9071. Gene and functional annotations were performed with PGAP [23] v4.13 and RASTtk [24] v2.0. Metabolic pathway analyses were performed using the KEGG database [25] Rel 93.0. CRISPRs were identified by CRISPRCasFinder [26], genomic islands were predicted by IslandViewer 4 [27], prophages were identified by PHASTER [28] and smBGCs were identified with antiSMASH [29] v5.1.2. All bioinformatics tools used for genome assembly and analyses were operated with default parameters or as specified in the text.

The complete genome of *P. fragi* A13BB comprises a single chromosome 4,940,458 bp in size with a GC content of 59.40%. Genomic features include 4410 CDSs, 25 rRNA, 73 tRNA, 4 ncRNA, 3 CRISPRs, 3 prophage regions and 37 predicted genomic islands (data file 8) [30]. Also, 353 subsystems comprising of various gene clusters including those associated with bioremediation, environmental adaptation, biocontrol, and plant growth promotion were identified (data file 9) [31]. Two β-lactone smBGCs, both showing low homology (20%) to known smBGCs, were identified. β-lactones are known for their antibiotic, anticancer and antiobesity properties [32]. A siderophore smBGC was identified even though *P. fragi* is considered a non-siderophore producing member of the genus *Pseudomonas* [33]. Arylpolyene and NAGGN smBGCs were also identified which, along with the siderophore smBGC, are likely to contribute to the environmental fitness of the strain [34–36]. Table 1 provides the links to data files 1–9.

**Table 1:** Overview of data files/data sets

Label	Name of data file/data set	File types (file extension)	Data repository and identifier (DOI or accession number)
Data file 1	Composition of ultra-minimal substrate growth medium	Portable Document Format file (.pdf)	10.6084/m9.figshare.12781193.v1 [7]
Data file 2	Quality distribution of Illumina reads	Portable Network Graphic file (.png)	10.6084/m9.figshare.13490967.v1 [11]
Data file 3	Basic quality statistics of GridION sequencing data	Portable Document Format file (.pdf)	10.6084/m9.figshare.13491147.v1 [13]
Data file 4	Average GridION read quality plot	Portable Network Graphic file (.png)	10.6084/m9.figshare.13491210.v1 [14]
Data file 5	Quast report	Portable Document Format file (.pdf)	10.6084/m9.figshare.13491228.v1 [17]
Data file 6	Short BUSCO summary	Portable Document Format file (.pdf)	10.6084/m9.figshare.13491234.v1 [19]
Data file 7	Assembly graph	Portable Network Graphic file (.png)	10.6084/m9.figshare.14370608.v1 [21]
Data file 8	Predicted Genomic Islands of *P. fragi* A13BB	Portable Document Format file (.pdf)	10.6084/m9.figshare.13491300.v1 [30]
Data file 9	Metabolic pathways of interest in *P. fragi* A13BB and associated gene clusters	Portable Document Format file (.pdf)	10.6084/m9.figshare.13507971.v1 [31]
Data set 1	Illumina and GridION sequencing reads	Fastq file (.fastq.gz)	https://identifiers.org/ncbi/insdc.sra:SRP251948 [37]
Data set 2	Genome assembly of *P. fragi* A13BB	Fasta file (.fna)	https://identifiers.org/insdc.gca:GCA_015767515.1 [38]

We believe the dataset presented in *Pseudomonas fragi* strain A13BB chromosome, complete genome [39] and in this data note form a sound basis for further in-depth study of the metabolic and biosynthetic capabilities of this strain, and indeed of other closely related species. The dataset also provides useful insights into the molecular mechanisms that underpin these capabilities. Furthermore, being only the fourth publicly available complete genome sequence of *P. fragi*, the data will enrich the comparative genomics study of the species.

### Limitations

IslandViewer 4 was run with default parameters. Crucially, IslandPick was run with default comparison genomes; different comparison genomes at different phyletic distances may influence the output of the analysis i.e. number of predicted genomic islands.

## Data Availability

Data files 1–9 described in this Data note can be freely and openly accessed on Figshare (https://figshare.com/) [7, 11, 13, 14, 17, 19, 21, 30, 31]. Datasets 1 and 2 can be freely and openly accessed on the NCBI database. Illumina and GridION reads generated have been deposited in the Sequence Read Archive under accession number SRP251948 (Dataset 1) [37]. The genome assembly of *P. fragi* A13BB has been deposited in GenBank under accession number GCA_015767515.1 (Dataset 2) [38]. The BioProject accession number for the entire project is PRJNA610978. Please see Table 1 and references for details and links to the data.

## Abbreviations

GC: Guanine-Cytosine
CDSs: Coding sequences
RNA: Ribonucleic acid
rRNA: Ribosomal ribonucleic acid
tRNA: Transfer ribonucleic acid
ncRNA: Non-coding ribonucleic acid
CRISPRs: Clustered regularly interspaced short palindromic repeats
PGPR: Plant growth-promoting rhizobacteria
smBGCs: Secondary metabolite biosynthetic gene clusters
DNA: Deoxyribonucleic acid
gDNA: Genomic deoxyribonucleic acid
PCR: Polymerase chain reaction
ONT: Oxford nanopore technology
ANI: Average nucleotide identity
NAGGN: N-acetylglutaminylglutamine amide
